# BRAF Activation Initiates but Does Not Maintain Invasive Prostate Adenocarcinoma

**DOI:** 10.1371/journal.pone.0003949

**Published:** 2008-12-16

**Authors:** Joseph H. Jeong, Zhenxiong Wang, Alexander S. Guimaraes, Xuesong Ouyang, Jose L. Figueiredo, Zhihu Ding, Shan Jiang, Isil Guney, Gyeong Hoon Kang, Eyoung Shin, William C. Hahn, Massimo F. Loda, Cory Abate-Shen, Ralph Weissleder, Lynda Chin

**Affiliations:** 1 Department of Medical Oncology, Dana-Farber Cancer Institute, Harvard Medical School, Boston, Massachusetts, United States of America; 2 Center for Systems Biology, Massachusetts General Hospital, Harvard Medical School, Boston, Massachusetts, United States of America; 3 Department of Urology and Pathology, Columbia University College of Physicians and Surgeons, Herbert Irving Comprehensive Cancer Center, New York, New York, United States of America; 4 Department of Pathology, Seoul National University College of Medicine, Seoul, Korea; 5 Department of Pathology, Brigham and Women's Hospital, Harvard Medical School, Boston, Massachusetts, United States of America; 6 Center for Cancer Genome Discovery, Dana-Farber Cancer Institute, Boston, Massachusetts, United States of America; 7 Department of Dermatology, Harvard Medical School, Boston, Massachusetts, United States of America; 8 Broad Institute of Harvard and MIT, Cambridge, Massachusetts, United States of America; 9 Center for Molecular Imaging Research, Massachusetts General Hospital, Harvard Medical School, Charlestown, Massachusetts, United States of America; 10 Department of Systems Biology, Harvard Medical School, Boston, Massachusetts, United States of America; University of Arkansas for Medical Sciences, United States of America

## Abstract

Prostate cancer is the second leading cause of cancer-related deaths in men. Activation of MAP kinase signaling pathway has been implicated in advanced and androgen-independent prostate cancers, although formal genetic proof has been lacking. In the course of modeling malignant melanoma in a tyrosinase promoter transgenic system, we developed a genetically-engineered mouse (GEM) model of invasive prostate cancers, whereby an activating mutation of BRAF^V600E^–a mutation found in ∼10% of human prostate tumors–was targeted to the epithelial compartment of the prostate gland on the background of Ink4a/Arf deficiency. These GEM mice developed prostate gland hyperplasia with progression to rapidly growing invasive adenocarcinoma without evidence of AKT activation, providing genetic proof that activation of MAP kinase signaling is sufficient to drive prostate tumorigenesis. Importantly, genetic extinction of BRAF^V600E^ in established prostate tumors did not lead to tumor regression, indicating that while sufficient to initiate development of invasive prostate adenocarcinoma, BRAF^V600E^ is not required for its maintenance.

## Introduction

Prostate cancer (PCA) is the most common malignancy affecting men over age of 65. Initially responsive to hormonal ablation therapy, PCA invariably recur and evolve to become lethal androgen-independent (AI) disease. While a number of common genetic events have been implicated in human prostate carcinogenesis including those targeting *PTEN*, *RB*, and *p27* tumor suppressors, *NKX3.1* tumor modulator, and the *c-Myc* oncogene[1], [2], [3], [4], [5], the genetic and biological basis governing progression to invasive and metastatic or AI disease is less well understood. Extensive genetic and experimental evidence have underscored the importance of the PI3K-PTEN-AKT signaling pathway, not only in genesis[3], [4], [5] but also in progression[6], [7] of PCA. In addition, specific genetic events, such as androgen receptor mutation or amplification, Bcl2 activation, and/or loss of p53 tumor suppressor function, had been associated with transition to AI disease[5], [8], [9], [10], [11], [12], [13], [14], [15], [16].

In contrast, the role of activated RAS-RAF-MAPK signaling in PCA is less well-established, although a growing body of evidence implicates the pathogenetic relevance of this pathway in prostate cancer biology. First, MAP kinase activation has been shown to correlate with disease progression in human PCA specimens[17]. Second, virtually all AI xenografts exhibit elevated phospho-MAP kinase levels and RAS activation renders LNCaP cells less dependent on androgens *in vitro* [18]. Furthermore, analysis of various RAS effecter mutants with differential capacity to engage specific downstream signaling pathways has also highlighted the MAPK axis in reducing androgen-dependence of LNCaP cells. Third, activating mutations in all three RAS family members have been reported in human PCA specimens[19], primarily from Japanese men, wherein these early studies reported a 13–30% frequency of mutations[20], [21], [22], [23], [24]. More recently, in a study of Korean patients, KRAS activating mutations were detected in 7% of cases and another 10% harbored the BRAF^V600E^ activating mutation[25]. In consonance with these mutation data, Raf1 expression is often found to be elevated and B-Raf inhibitor reduced in human prostate tumors[26]. Lastly, ETV1/ER85, a partner in the high frequency TMPRSS2:ETV1 chromosomal fusion event in human prostate cancer, is a downstream target of RAS-RAF-MAPK signaling[27]. These reinforcing, albeit correlative, data have implicated activated RAS-RAF-MAPK signaling in the prostate cancer genesis and progression.

Genetically engineered mouse (GEM) models have enabled the validation and functional analysis of several key genetic alterations found in prostate cancer development (reviewed in[28], [29]. The majority of invasive PCA GEM models have largely emphasized AKT activation strategies such as *myr-AKT* or *Pten* deletion, alone or together with *p53* or *p27* inactivation[3], [4], [5], [12], [30], [31], [32]. Other well-established models include prostate-specific expression of *c-Myc* or SV40 oncogenes[1], [33], [34]. GEM model of invasive prostate cancers driven by MAPK activation has not been reported.

Previously, we have engineered an inducible bitransgenic HRAS^V12^-driven melanoma model possessing both activator (Tyr-rtTA) and reporter (Tet-HRAS^V12^) transgenes on *Ink4a/Arf*−/− background (hereafter designated as “iHRAS*”)[35]. The rtTA transgene was driven by a Tyrosinase enhancer-promoter element consisting of a 5 Kb upstream enhancer element fused to the proximal promoter of mouse tyrosinase gene[36]. This promoter has been used extensively as a melanocyte-specific promoter element[35], [36], [37]. To our surprise, while majority of the iHRAS* mice succumbed to invasive and angiogenic cutaneous melanomas in a doxycycline-dependent manner[35], a handful of the aging male mice that escaped the melanoma fate developed invasive prostate tumors (unpublished observations). Similarly, male mice from an identically engineered iNRAS* model also succumbed to prostate cancer if they did not develop melanoma (unpublished observations). These unanticipated observations suggest that prostate cancer is a late-onset tumor phenotype in RAS-activated GEM models engineered with this particular tyrosinase promoter-enhancer element. Furthermore, it raised the possibility that oncogenic alleles with weaker activity in melanocytes might favor more robust prostate cancer phenotype.

Indeed, when we constructed an iBRAF* model, the iBRAF* mice were minimally melanoma-prone (data not shown). Instead the iBRAF* transgenic males were highly susceptible to the development of aggressive prostate neoplasms in a doxycycline-dependent manner. Since activated BRAF is one of the most potent activators of MAP kinase signaling and BRAF^V600E^ mutation itself has been described in a subset of human prostate cancers[25], this iBRAF* model represents a potentially useful *in vivo* system in which to address the role of MAP kinase activation in prostate cancer genesis and progression.

## Results

### BRAF* activation drives aberrant proliferation in p63+ basal epithelial cells of the prostate

**Figure 1:**
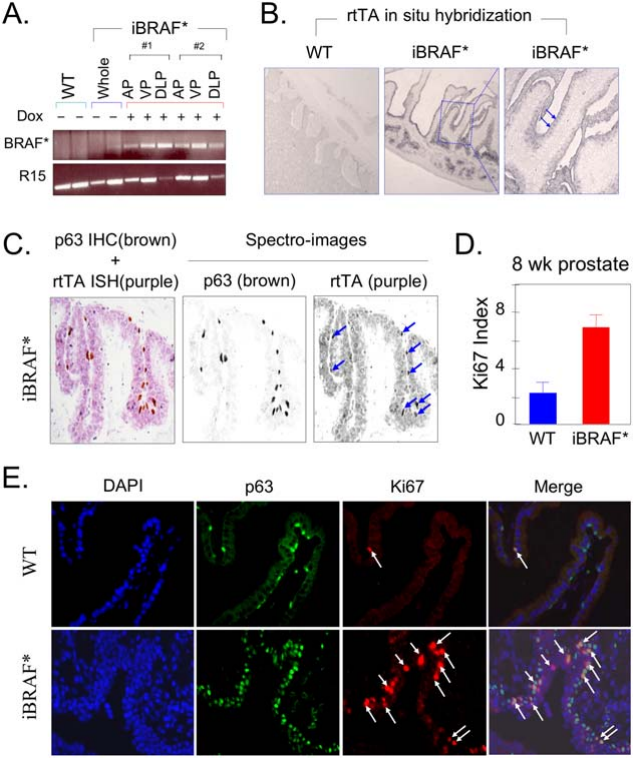
Transgene BRAF* expression in prostate epithelium drives aberrant proliferation of the p63+ prostatic basal cells. A. Transgene BRAF* transcript was detected in all three lobes (AP, anterior prostate; VP, ventral prostate; DLP, dorsolateral prostate) of the prostate glands from two independent 8-week-old bi-transgenic iBRAF* male mice on doxycycline by transgene-specific RT-PCR. As controls, whole prostate glands were isolated from WT or iBRAF* off doxycycline mice (n = 2 for each). Ribosomal protein R15 was used as an internal control for RT-PCR. B. RNA *in situ* hybridization (RISH) using rtTA riboprobe documented expression of BRAF* transgene in prostate epithelial gland (200×). Arrows indicate the expression of *rtTA* in luminal cell compartment in zoom-in with higher magnification (400×). C. Expression of BRAF* transgene was detected in both luminal cells and p63+ basal cells of the prostate epithelium of 8-week-old bi-transgenic iBRAF* male mice on doxycycline using dual serial staining of p63 IHC and rtTA RISH. After quick incubation of slides with antibody against p63 for 10 minutes with IHC procedure, RISH procedure with rtTA RNA probe was followed. Brown color for p63+ basal cells by IHC and purple color for *rtTA* expression by RISH were differentially detected by spectro-imaging machine. Note the co-localization of strong *rtTA* expression and p63 immuno-reactivity in approximately 50% of p63+ basal cells (arrows). D. Ki67 staining of histologically-normal prostate glands in 8-week-old males showed increased proliferation index in iBRAF* transgenic (on doxycycline) compared to WT. E. Co-immunofluorescence study in prostate glands isolated from 8-week-old WT and iBRAF* transgenic (on doxycycline) males showed the expansion of p63+ basal cells in the prostate glands of iBRAF* mice. Approximately half of the p63+ cells were in proliferation as measured by co-staining with Ki67 (see arrows).

The occurrence of prostate cancers in tyrosinase promoter/enhancer-driven transgenic mice prompted a detailed analysis of transgene expression in the prostate. Examination of transgene expression was performed in all 3 lobes of the prostate glands in 8-week-old bi-transgenic iBRAF* males (iBRAF*, short for “Tyr-rtTA::Tet-BRAF^*^::Ink4a/Arf−/−”). We detected BRAF* transcripts in all 3 lobes of iBRAF* mice on doxycycline (n = 2 mice examined), but not in whole prostate glands derived from WT or iBRAF* transgenic males off doxycycline using transgene-specific RT-PCR (Figure 1A; n = 2 for each). As RNA *in situ hybridization* (RISH) of BRAF detects both endogenous *BRAF* and transgene BRAF* expression throughout the prostatic epithelium (data not shown), we tracked transgene BRAF* expression by *rtTA* RISH (*nb*., *rtTA* is foreign to the mammalian genome). Upon doxycycline exposure, rtTA is known to potently drive expression of a transgene linked to a Tet-responsive promoter and is therefore a highly specific read-out of the Tet-driven transgene (BRAF* in this case) expression. *rtTA* transcripts were not detected in non-transgenic wild-type prostate gland, yet were abundant in iBRAF* transgenic prostates exposed to doxycycline (Figure 1B), particularly in the luminal cells (Figure 1B, zoom-in panel, see arrows). To further refine localization of transgene expression in the epithelium and determine whether the transgene is also expressed in the basal cell compartment, we performed serial p63 IHC and *rtTA* RISH (see Materials & Methods) in 8-week-old male iBRAF* prostate glands induced on doxycycline (Figure 1C). Indeed, *rtTA* transcripts were found in both p63+ basal cells and luminal cells (approximately 50% of the p63+ basal cells showed stronger *rtTA* expression; see arrows), indicating that this transgenic system can target expression to both the basal progenitor and the luminal compartments of prostate glands.

To examine the functional impact of BRAF* transgene expression in the prostate, we monitored doxycycline-dependent proliferative responses in histologically normal prostates of iBRAF* and control mice. These analyses revealed enhanced epithelial proliferation documented by increased Ki67 index from 2.25 (+/−0.83) per 100 nuclei in 8-week-old WT to 6.9 (+/−0.95) per 100 nuclei in age-matched iBRAF* prostate (n = 300 nuclei/area×5 areas counted in each sample; p = 0.00035; Figure 1D). Notably, this BRAF*-induced proliferative response was more prominent in the basal cell compartment, as evident by the observation that most of the strong Ki67 positive signals were confined to p63+ cells (Figure 1E; approximately 50% of p63+ basal cells in iBRAF* prostate glands were positive for Ki67).

### iBRAF* mice developed invasive adenocarcinoma of the prostate

**Figure 2:**
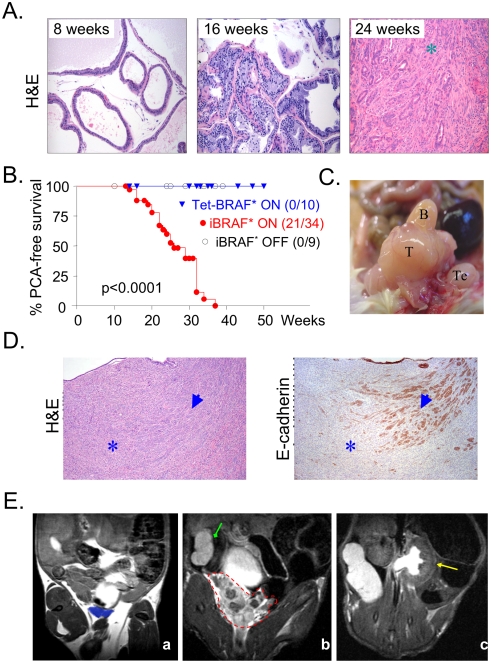
iBRAF* transgenic males develop invasive adenocarcinoma of the prostate. A. Time-course histological analysis of prostate samples from iBRAF* transgenic mice (on doxycycline) at the indicated ages. Hyperplastic lesions were detected in a 16-week-old iBRAF* transgenic mouse on doxycycline. Up to an age of 24 weeks, approximately 50% of iBRAF* transgenic mice developed PCA. B. Kaplan-Meier analysis of PCA-free survival in iBRAF* (on doxycycline, n = 34), iBRAF* (off doxycycline, n = 9), and Tet-BRAF* (on doxycycline, n = 10) transgenic males. Only double transgenic iBRAF* mice given doxycycline in their drinking water developed PCA with an average latency of 24 weeks (standard deviation = ±6 weeks). C. Gross morphology of a prostate tumor from an iBRAF* transgenic mouse. T indicates tumor; B, bladder; and Te, testis. D. The transition from ductal (arrowhead) to spindle (asterisk) morphology is coincident with loss of E-cadherin expression in iBRAF* PCA, consistent with EMT (epithelial-mesenchymal transition). E. Detection of PCA in live mice by MRI. Panel a is a representative image of a WT mouse with normal prostate (highlighted in blue). Panel b is a representative MR image of PCA in an iBRAF* male on doxycycline (red outline; note heterogeneous signals within the lesion). Panel c is a representative MR image of hydronephrosis detected in an iBRAF* mouse with PCA. Note enlarged distended kidney (yellow arrow) in contrast to normal kidney in panel b (green arrow), indicating accumulation of urine in the kidney due to outflow obstruction by the tumor.

A serial histopathological examination of the prostates from iBRAF* transgenic males was conducted to assess the long-term consequences of sustained BRAF* expression in the prostate epithelium. After 5 weeks of iBRAF* induction in 8-week-old animals (left panel in Figure 2A), the prostate gland appeared largely normal although moderate degree of aberrant proliferation was already evident (Figure 1E). Basaloid hyperplasia became evident in 16-week-old mice (middle panel in Figure 2A), consistent with prominent proliferative responses in the basal compartment (Figure 1E), followed by emergency of frank adenocarcinoma by 24 weeks of age (right panel in Figure 2A). Careful follow-up and characterization of a large colony of iBRAF* and control mice showed that only iBRAF* males on doxycycline were prone to PCA development with high penetrance. In founder line #29, 21/34 bi-transgenic iBRAF* males on doxycycline (ON) developed prostate tumors with an average latency of 24 weeks (SD = ±6 weeks) (Figure 2B, Supporting Table S1). In comparison, all iBRAF* mice off doxycycline (OFF, n = 10) and single transgenic Tet-BRAF* mice (e.g. Tet-B-RAF*::Ink4a/Arf−/−) on doxycycline (ON, n = 9) remained PCA free (Supporting Table S1). Similar observations were made in a smaller cohort derived from founder line #13 where 3/10 doxycyline-treated iBRAF* males developed PCA with a latency of 23±6 weeks (Supporting Table S1). Additionally, BRAF* transgene expression could be documented in these PCA tumors by RT-PCR and RISH (Supporting Figure S1; data not shown for RISH).

iBRAF* PCA tumors were rapidly growing, reaching large size and causing local obstruction (Figure 2C). Consistent with its aggressive nature, iBRAF* tumors exhibited epithelial-mesenchymal transition (EMT) associated with downregulation of E-cadherin at the transition from ductal to spindle morphology in these tumors (Figure 2D, compare asterisk to arrow). By high-spatial resolution MRI, PCA could be readily discerned by the appearance of an enlarged, heterogeneous mass often compressing the bladder (Figure 2E-b, dashed outline; see panel E-a for normal prostate size marked by blue highlight). As in some human patients, these large lesions caused bladder outlet obstruction and hydronephrosis–distention of the kidney due to outflow obstruction (Figure 2E-c yellow arrow).

The complex and context-specific interactions between RAS and PI3K signaling components coupled with the well-documented prominence of PI3K-PTEN-AKT aberrations in PCA prompted analysis of downstream signaling events in a collection of iBRAF* PCA tumors. Consistent with the known ability to BRAF* to potently activate MAPK signaling, all iBRAF* tumors showed p-ERK activation by Western blot and IHC analyses (Figure 5A,B). Notably, while Western blot analysis of *Pten*-null PCA showed strong AKT activation, none of the iBRAF* tumors examined showed AKT activation (Figure 5A). IHC analysis of additional iBRAF* prostate tumors (n = 17) mirrored these Western blot results, showing absence of AKT activation and readily detectable p-S6 immunoreactivity (Figure 5C). Thus, activated BRAF* expression targeted to prostate epithelium in the context of *Ink4a/Arf* deficiency triggers a proliferative response in the basal p63+ compartment, which when sustained, progresses with high penetrance to basaloid hyperplasia and ultimately invasive adenocarcinoma, without evidence for concomitant AKT activation.

### iBRAF* prostate tumors are epithelial

**Figure 3:**
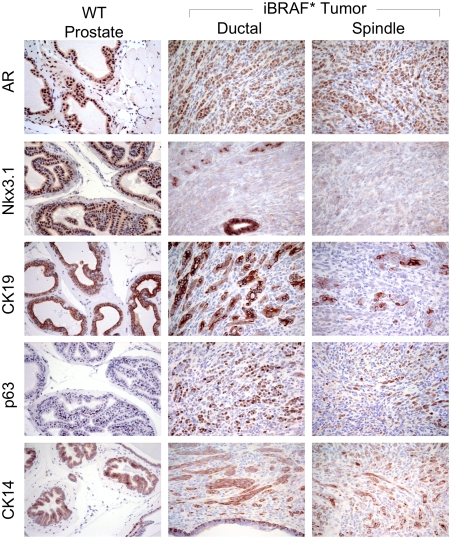
iBRAF* prostate tumors exhibit epithelial lineage markers. Both ductal and spindled components of iBRAF* prostate tumors express epithelial lineage markers: CK14 and p63 for basal cell, AR and Nkx3.1 for luminal cell, and CK19 for transit-amplifying cells.

Given the complex histopathological presentation of the tumors, we utilized a suite of validated prostate lineage markers to determine the origin of these iBRAF* tumors. As shown in Figure 3, androgen receptor (AR) and Nkx3.1 are expressed in both ductal and spindled components. Nkx3.1 is the earliest known differentiation marker of the prostate luminal epithelium[8], and interestingly its expression was significantly reduced or absent in the spindled cell region of the tumors, possibly reflecting a less differentiated state. Basal cell markers, cytokeratin (CK) 14 and p63 were strongly positive, especially in the ductal components, as was CK19, a marker for transit-amplifying or intermediate differentiated cell[38], [39]. This epithelial markers profile, coupled with negative immunoreactivity to chromogranin and synaptophysin, two markers of neuroendocrine cells[40] (Supporting Figure S2), indicates that iBRAF* PCA are epithelial tumors.

**Figure 4:**
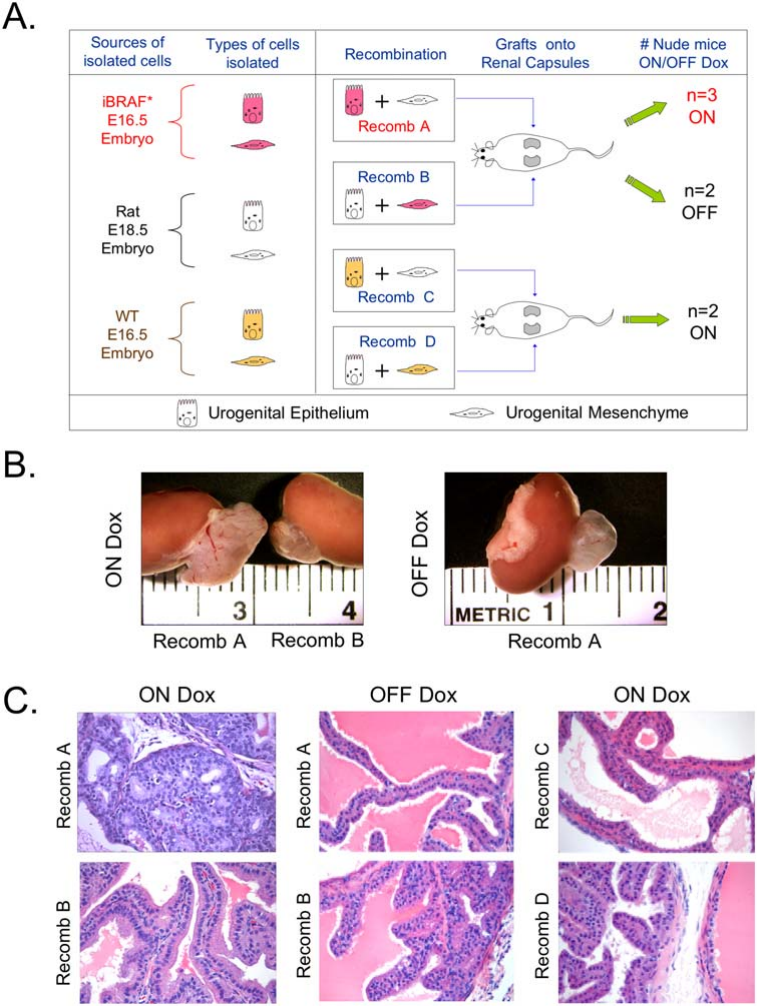
iBRAF* embryonic urogenital epithelium drives basaloid hyperplasia in the tissue recombination system. A. Schematic representation of the tissue recombination protocol. Recombinants of iBRAF* transgenic mouse epithelium and rat mesenchyme (Recomb A) were grafted under the right kidney capsules of adult nude mice. The converse recombinants (iBRAF* transgenic mouse mesenchyme and rat epithelium; Recomb B) were also transplanted in the same manner into the left kidney capsule of the same mice. Three of the grafted mice were fed doxycycline drinking water, and two were not. As controls, recombinants of non-transgenic wild-type mouse epithelium and rat mesenchyme (Recomb C) and the converse recombinants (Recomb D) were grafted in the same manner. Grafts were harvested 6 weeks later. B. Macroscopic images show that grafts with Recomb A on doxycycline are larger than both grafts with Recomb B on doxycycline and grafts with Recomb A off doxycycline. C. Histological analysis of these tissue grafts confirmed basaloid hyperplasia only in grafts with Recomb A on doxycycline, while others showed normal histology.

**Figure 5:**
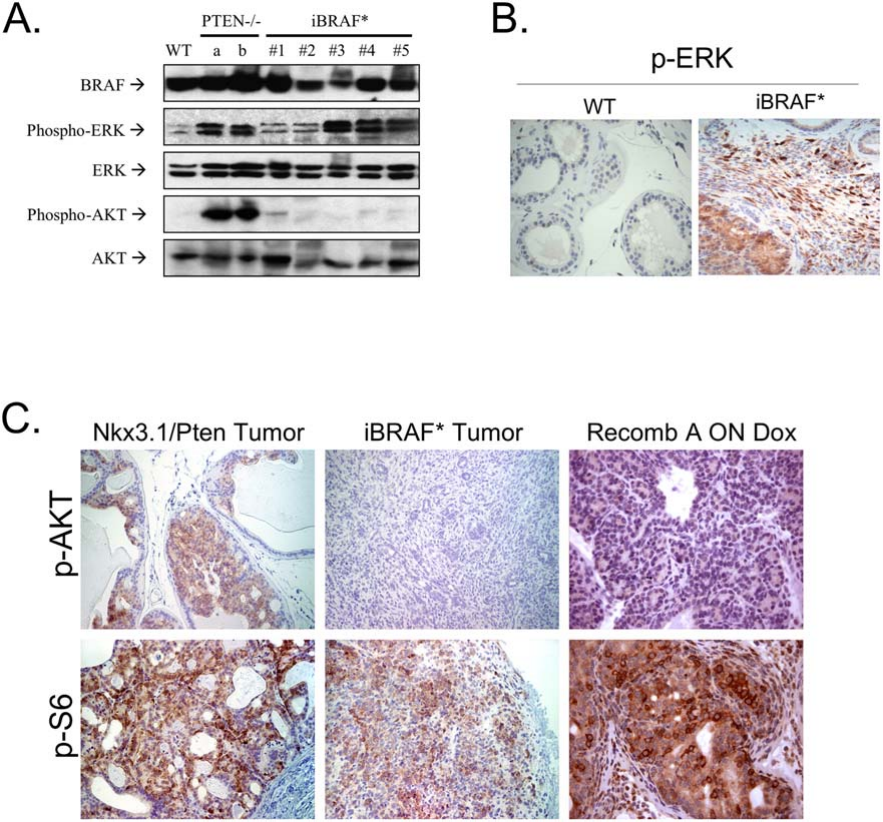
iBRAF* prostate tumors are AKT-independent. A, B. p-ERK activation in iBRAF* PCA tumors (n = 5; on doxycycline) was detected by immunoblotting analysis (A) and IHC (B). In contrast, p-AKT activation was not detected in all iBRAF* PCA tumors (n = 5; on doxycycline). Controls (a & b) were two prostate cancer samples from *Pten*-null mice and WT was prostate glands from a WT mouse. C. p-AKT immunoreactivity was not detected on iBRAF* PCA tumors (n = 17) and Recomb A (a recombinant of iBRAF* transgenic mouse epithelium and rat mesenchyme on doxycycline) by IHC, but strong immunoreactivity of p-S6 was still present. PCA sample from *Nkx*3.1−/−, *Pten*+/− compound mutant mice were used as control.

To functionally document the epithelial nature of iBRAF* PCA, we utilized the tissue recombinant approach pioneered by Cunha and colleagues[41]. Here, recombinant of epithelium from iBRAF* or wild type mice was mixed with mesenchymal tissues from mice or rats (Figure 4A for design) then grafted under the kidney capsules of adult nude male mice. Grafts were harvested 6 weeks later. As shown in Figure 4B, Recomb A grafts (comprising iBRAF* epithelium and rat mesenchyme) were significantly larger than Recomb B grafts (the converse) from nude mice supplemented with doxycycline drinking water (Figure 4B left panel). On the other hand, without doxycycline, Recomb A grafts were normal in size (Figure 4B right panel). Histological analysis of these grafts confirmed basaloid hyperplasia and PIN in grafts from Recomb A ON doxycycline only, while Recomb B grafts from the same hosts or Recomb A from hosts without doxycycline showed normal glandular structure that was indistinguishable from the Recomb C and D controls (Figure 4C). These doxycyline-induced hyperplastic and PIN lesions from Recomb A ON doxycycline grafts exhibited similar marker profile as described for tumors from the *de novo* transgenic mice-positive for Nkx3.1, AR, CK19, CK14 and p63 (Supporting Figure S3; data not shown for CK19 and CK14) as well as evidence for significant expansion of the p63+ compartment (Supporting Figure S3). On the biochemical level, a profile of robust p-ERK and p-S6K activation without detectable p-AKT activity was observed in these tissue recombinant specimens similar to tumors from *de novo* transgenic mice (Figure 5C right panel and data not shown for p-ERK). In summary, lineage marker characterization of *de novo* transgenic tumors, coupled with the functional data in the tissue recombinant system, provides clear evidence that iBRAF* PCA are invasive adenocarcinomas expressing luminal, intermediate and basal cell markers.

### iBRAF* PCA progress to indolent androgen-independent tumors after castration

**Figure 6:**
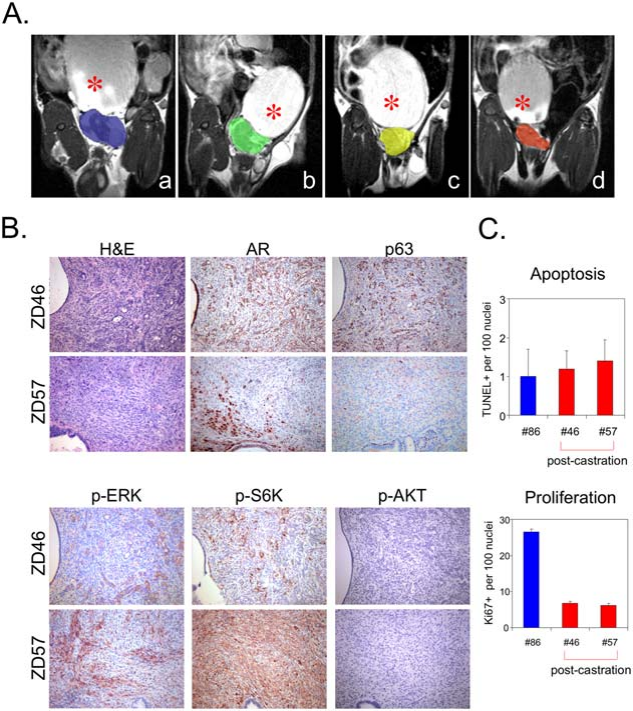
iBRAF* PCA progress to indolent androgen-independence after castration. A. The reduction of tumor volumes was monitored by MRI over a 4-week time course after castration (a, tumor (blue) 1 week after castration; b, tumor (green) 2 weeks after castration; c, tumor (yellow) 3 weeks after castration; d, tumor (orange) 4 weeks after castration). Asterisk indicates bladder. B. Histological examination confirmed the presence of prostatic tumor cells in two castrated iBRAF* mice (#46 and #57). The two post-castration tumors were positive for AR (a prostatic luminal cell marker) and p63 (a basal cell marker) by IHC. Also, the post-castration tumor cells were negative for p-AKT, but remained strongly positive for p-ERK and p-S6K on IHC. C. The post-castration tumor cells were viable and growing, not residual tumor remnants, as manifested by low apoptosis index (1.2 (+/−0.45) and 1.4 (+/−0.55) per 100 nuclei for #46 and #57, respectively) on TUNEL, comparable to pre-castration tumor (1.0 (+/−0.71)) (top), and active proliferation by Ki67 staining, albeit at rate much lower than that of pre-castration tumor (6.8 (+/−3.6) and 6.2 (+/−2.2) for #46 and #57, respectively and 26.6 (+/−3.6) in the pre-castrated tumors) (bottom).

Emergence of androgen-independence represents the most significant disease process in human PCA from the standpoint of mortality, prompting us to determine whether the iBRAF* model develops androgen independent (AI) tumors. To this end, we identified 10 iBRAF* mice with documented PCA by MRI screening for heterogeneous mass in the prostate gland (representing PCA) for castration. Despite frequent bladder irrigation in an effort to relieve outlet obstruction, 50% (n = 5) animals succumbed peri-operatively which was likely due to a generally compromised physiological state secondary to renal failure. Of the surviving animals, two were sacrificed at one-week post-castration (#36 and #57) and the remaining 3 were followed by weekly MRI for 4 weeks. Volumes of the prostate mass in all 5 mice decreased during the observation period (Figure 6A and Supporting Figure S4) but histopathological examination identified residual tumor nodules in two of the five castrated iBRAF* mice (#46 and #57). These tumor nodules were AR+ and p63+ with strong p-ERK and p-S6K activation but without detectable p-AKT (Figure 6B). That they were comprised of viable malignant cells was shown by low apoptosis index, comparable with index observed in pre-castrated tumor (Figure 6C). However, compared to the rapidly growing pre-castration tumors, these post-castrated tumors were indolent, with proliferation indices of 6.8 (+/−3.6) and 6.2 (+/−2.2), respectively, compared to an index of 26.6 (+/−3.6) in the pre-castrated tumors (n = 300 nuclei/area×5 areas counted in each sample; p<0.001 for both; Figure 6C). Thus, it appears that BRAF driven ERK and S6K activation alone is not sufficient to drive androgen-independent growth post castration, although it appears to be permissive of survival in low androgen state.

### BRAF activation is not required for iBRAF* PCA maintenance

**Figure 7:**
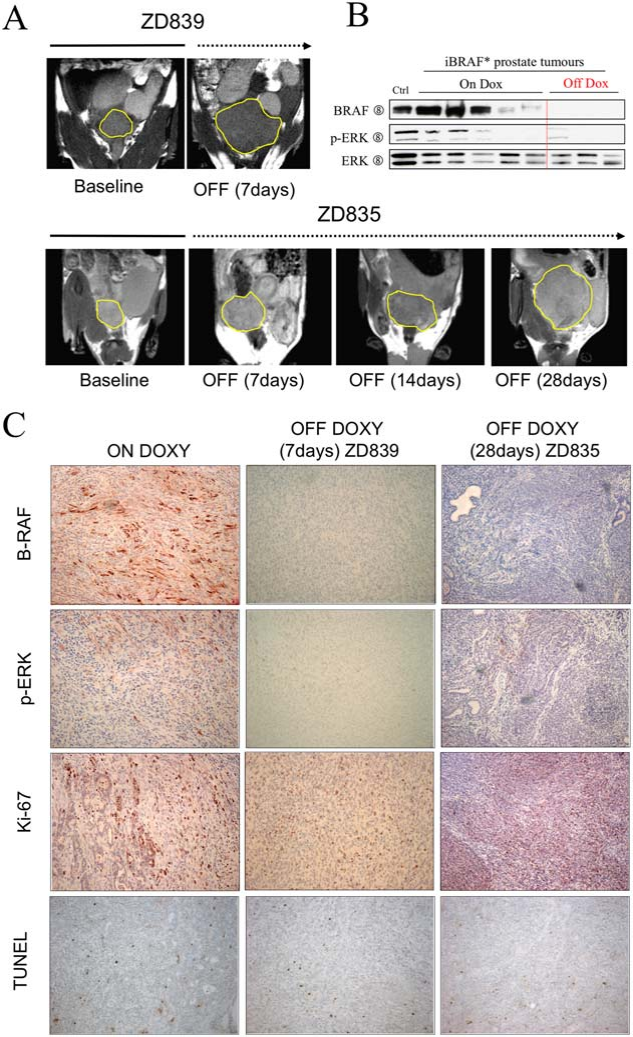
iBRAF* prostate tumors do not require BRAF activation for their tumor maintenance. A. Tumor size change was monitored by serial MRI imaging before (baseline; solid line) and after (indicated periods; dotted line) doxycycline withdrawal (7 days for ZD839 and 28 days for ZD835). Yellow line indicates tumor boundary. B. B-RAF expression and p-ERK activation were not detected by immunoblotting analysis in most of off doxycycline iBRAF* PCA tumors (n = 3), although one of the off doxycycline iBRAF* tumor samples (ZD835) showed weak p-ERK activation. C. Immunohistological examination using antibodies against B-RAF and p-ERK confirmed the repression of B-RAF* transgene repression during off doxycycline periods (7 days for ZD839 and 28 days for ZD835). However, the tumors were viable and still growing, as manifested by low apoptosis on TUNEL and active proliferation by Ki67 staining.

To determine whether constitutive BRAF* signaling is required for iBRAF* tumor maintenance, we performed doxycycline withdrawal study in iBRAF* transgenic animals with documented PCA by MRI or by physical examination. First, two iBRAF* mice were identified as tumor-bearing and enlisted into serial MRI after doxycycline was removed from the drinking water. Unexpectedly, tumors continued to grow in both animals as shown on MRI (Figure 7A), requiring termination and sacrifice of ZD839 at one week and ZD835 at 4 weeks post doxycycline withdrawal. Similarly, two additional iBRAF* mice with tumors by palpation were taken off doxycycline; close follow-up revealed continued tumor growth, requiring sacrifice at 2 weeks and 4 weeks, respectively (data not shown). These off-doxycycline tumors did not express BRAF* by Western blot analysis (Figure 7B) or by IHC (Figure 7C, compare immunoreactivity to left panel). Accordingly, ERK phosphorylation was undetectable in ZD839 and only patchy in ZD835. Consistent with its continued growth *in vivo*, robust Ki67 and minimal TUNEL staining was documented in these off-doxycycline tumors, similar to the profile of on-doxycycline iBRAF* PCA. Taken together, these observations indicate that, while sustained BRAF* activation in the prostate gland is sufficient to drive development of invasive prostate adenocarcinoma, it is not required for maintenance of established PCA.

## Discussion

This study, together with recently reported BRAF mutations in human prostate tumors[25], demonstrates the pathogenetic relevance of MAP kinase activation in prostate tumorigenesis. By targeting BRAF V600E–a human-relevant mutation known to potently activate MAP kinase–to the mouse prostate epithelium, we show here that MAP kinase activation can drive aberration proliferation and basaloid hyperplasia, leading to emergence of invasive adenocarcinoma of the prostate gland with short latency and high penetrance without evidence of AKT activation. The profile of strong ERK and S6K activation in the absence of AKT in iBRAF* tumors is consistent with the model proposed by Pandolfi and colleagues, whereby constitutive ERK activation inhibits TSC complex and subsequent activation of mTOR and downstream S6K[42]. In a cohort of four BRAF^V600E^ mutated human PCA with strong pERK and pS6K activation by IHC, weak to absent pAKT immunoreactivity was indeed observed in two (#S04-7014 and #S04-7989 in Supporting Figure S6), supporting the notion that this iBRAF* transgenic system is modeling a subset of human PCA.

In mouse and human systems, activation of both AKT and ERK signaling is commonly observed during the initiation and progression of PCA[26], [43]. Since most mouse prostate cancer models are driven by AKT-activation, independent contribution of MAPK activation to prostate tumorigenesis has been difficult to establish. Here, by demonstrating that evolution from benign hyperplasia to invasive prostate tumors without concomitant AKT activation, this iBRAF* model offers the first genetic proof that MAPK activation alone is sufficient for initiation and progression to invasive PCA *in vivo*. Additionally, the ability to enforce MAPK activation via mutant BRAF expression under an androgen-insensitive promoter renders this iBRAF* model an ideal system for genetic dissection of MAPK contribution independent of AKT during AI progression. In this regard, although they grow rapidly in androgen-rich conditions, these AKT-negative iBRAF* PCA undergo complete or significant regression upon castration. Those indolent lesions surviving castration remain pAKT negative, pointing to its dispensability for survival in low-androgen state but likely requirement for AI growth *in vivo*, in line with recent study in *ex-vivo* manipulated system[26]. In summary, we conclude that while it may be permissive for survival post castration, BRAF driven MAPK activation is not sufficient to drive active growth under an androgen-limited state.

Finally, while MAPK activation alone can initiate prostate tumorigenesis, continued mutant BRAF expression or downstream MAPK activation is not required for maintenance of established PCA. Genetic inactivation of BRAF* by doxycycline withdrawal in 4 BRAF*-driven tumors did not lead to tumor growth inhibition nor regression. Corroborating with this was an intriguing finding that pharmacological inhibition of MEK with CI-1040 in renal capsule grafts of iBRAF* tumors (n = 2) did not inhibit tumor growth despite extinction of pERK activities (Supporting Figure S5). Taken together, these observations indicate that continued expression of mutant BRAF or activation of MAPK is not required to sustain growth or maintain viability of established PCA. This contrasts with findings in BRAF*-driven lung adenocarcinoma model where BRAF* acts not only as an initiating oncogene but shown to be required for maintenance[44], highlighting the context/lineage specific role(s) of an oncogenic event in genesis, progression and maintenance.

In summary, we describe here a novel genetically engineered mouse model of invasive PCA driven by MAPK activation via inducible BRAF mutation under an androgen-insensitive promoter. This model serves as a unique system for dissecting contribution of MAPK, relative to AKT, in development, progression and treatment of PCA.

## Materials and Methods

### Generation of inducible BRAF* (iBRAF*) transgenic mice

A human wild-type BRAF cDNA was cloned into a pBKS plasmid with its expression under the control of a minimal promoter containing multimerized tet-operons. A constitutively active form of mutant BRAF^E600^ was generated from the pBSK plasmid using QuikChange® Site-Directed Mutagenesis Kit (Stratagene) (designated Tet-BRAF^*^). The Tet-BRAF^*^ construct was injected into oocytes derived from *Ink4a/Arf* null mice. Transgenic mouse lines harboring the Tet- BRAF^*^ elements were crossed with another transgenic mouse lines expressing the reverse tetracycline transactivator under the control of the tyrosinase promoter/enhancer elements (designated Tyr-rtTA) to produce cohorts of single (Tet-BRAF*) and double (designated iBRAF*, Tet-BRAF*; Tyr-rtTA) transgenic animals. Doxycycline-supplemental drinking water was administered to induce transgene BRAF^E600^ expression as previously described[35].

All animal experiments were performed according to a protocol approved by the Institutional Animal Care and Use Committee (IACUC) at Harvard Medical School.

### Transplantation of tissue recombinants

A two-way tissue recombination was performed. Mouse and rat embryonic urogenital sinuses were obtained at 16.5 days post coitum (dpc) and 18.5 dpc, respectively, as described previously[41]. After treatment of trypsin, epithelium and mesenchyme were separated under the microscope. Next, mouse urogenital sinus epithelium (mUGE) and rat urogenital sinus mesenchyme (rUGM), or mouse urogenital sinus mesenchyme (mUGM) and rat urogenital sinus epithelium (rUGE), were combined. Tissue recombinants were grafted under the kidney capsules of adult male nude mouse host for 6 weeks (with doxycycline on or off). Upon harvesting the grafts, tissues were fixed in 10% formalin overnight, and processed for histology and immunostaining.

### Histology and immunohistochemistry

All the tissue samples were fixed in 10% formalin overnight and embedded in paraffin. Immunohistochemistry were performed as described previously[45]. For antigen retrieval, slides were heated in 0.01 M citrate buffer (pH 6.0) in the microwave four times at four minutes each time. The antibodies and dilutions are: BRAF, 1∶250 (Santa Cruz); phospho-Erk, 1∶250 (Cell Signaling); Androgen Receptor, 1∶250 (ABR); Ki-67, 1∶2000 (Novacastra); Cytokeratin-14, 1∶ 50 (Biogenex); p63, 1∶600 (Santa Cruz); Chromogranin A, 1∶4000 (Diasorin); Synaptophysin, 1∶500 (Santa Cruz); phospho-p70 S6 kinase 1∶250 (Cell Signaling); E-cadherin, 1∶250 (transduction lab); phospho-AKT(Ser473), 1∶100 (Cell Signaling); Nkx3.1, 1∶6000 (kindly provided by Dr. Cory Abate-Shen); and Cytokeratin 19, 1∶20 (kindly provided by Dr. Nabeel Bardeesy). Apoptotic cell death was detected using the ApopTag® Plus Peroxidase *In Situ* Apoptosis Detection Kit (Chemicon).

### RNA In-situ hybridization (RISH)

The 10% formalin-fixed, paraffin-embedded slides were used for in situ hybridization with DIG-labeled riboprobe. DIG-labeled RNA probes were synthesized from a pBKS plasmid containing a rtTA PCR product (500 bp) using either T7 (for sense probe) or T3 (for anti-sense probe) promoter by in vitro transcription system with DIG RNA Labeling Mix (Roche Molecular Biochemicals, Mannheim, Germany). After deparaffinization, slides were digested in proteinase K solution (50 ug/ml) for 10 minutes at 37°C. DIG-labeled rtTA RNA probes were diluted in hybridization buffer at the concentration of 1 ug/ml. 100 ul of the diluted RNA probes was added on each slide and covered by 24×40 mm^2^ coverslip. Slides were hybridized at 60°C overnight, and washed at 65°C for 15 minutes twice in 2× SSC buffer with gentle agitating. After the treatment of RNAse A (10 ug/ml) for 30 minutes at 37°C, the slides were washed for 10 minutes twice at room temperature (RT) in 2× SSC buffer and followed by additional washing for 30 minutes twice at 65°C in 0.2× SSC buffer with gentle agitation. The slides were washed in PBS for 15 minutes twice at RT and then incubated with anti-Digoxigenin antibody conjugated with alkaline phosphatase (1∶2000, Roche) overnight at 4°C. Color was developed by dipping the slides in NTB/BCIP solution[46], [47] approximately for two to four hours in the dark.

For the dual staining, first with immunohistochemical staining procedure, the quick incubation of the slides with antibody against p63 for 10 minutes was performed, and then followed by RNA in situ hybridization procedure with rtTA RNA probe as mentioned above.

### MRI methods

Magnetic resonance imaging was performed on a 4.7 T on a Bruker imaging system (Pharmascan, Karlsruhe, Germany). Protocols included a Tri-plane and coronal proton density weighted localizer. Multi-slice T2-weighted imaging was performed in the coronal and axial planes utilizing the following parameters: Flip angle = 90°; Matrix size (256×256); TR = 2500 msec; TE = 44.6 ms; field of view (FOV) = 4.24×2.12 cm, slice thickness = 1.2 mm. T1-weighted imaging was performed in the coronal and axial planes following the administration of intraperitoneal Gd-DTPA utilizing the following parameters: Flip angle = 90°; Matrix size (256×256); TR = 700 msec.; TE = 14 msec.; field of view (FOV) = 4.24×2.12 cm, slice thickness = 1.2 mm. Tumor volumes were determined by region of interest (ROI) analysis of T1-weighted post Gd-DTPA enhanced images using robust image analysis software (Osirix®). The sum of the region of interests was multiplied by the slice thickness to obtain tumor volumes. Tumor volumes are reported in cubic centimeter (cc).

### Molecular analysis

RNA was isolated from the prostate tumor samples, and reverse-transcribed to cDNA as described previously[45]. A RT-PCR primer pair for the detection of transgene-specific BRAF expression was as follows (210-bp fragment):

RTM-BRAF-5F: 5′-TCTTCATGAAGACCTCACA-3′

RTM-BRAF-5R: 5′-ACTGTCCAGTCATCAATTCA-3′

PCR amplification condition was 95°C for 15 min followed by 95°C for 1 min, 62°C for 1 min, and 72°C for 1 min with 31 cycles. As an internal control for RT-PCR, ribosomal protein R15 expression was used.

For western blot analysis, tumor lysates were extracted as described previously[45]. Total 20 micrograms of lysate was run on 4–12% Bis-Tris NuPAGE (Invitrogen), transferred to PVDF membrane (Perkinelmer), and blotted using the following antibodies: p-ERK, 1∶500 (Cell Signaling); phospho-S6 Kinase, 1∶500 (Cell Signaling); and p-AKT(Ser473), 1∶250 (Cell Signaling).

## Supporting Information

Table S1 (0.03 MB RTF)

Figure S1 BRAF* transgene expression is documented by transgene-specific RT-PCR with two independent prostate tumors of bi-transgenic iBRAF* mice on doxycycline.
(1.37 MB TIF)

Figure S2 Both ductal and spindled components of iBRAF* prostate tumors were negative for neuroendocrine markers (chromogranin and synaptophysin) by IHC. For controls, pancreatic tissues were used.
(1.37 MB TIF)

Figure S3 Grafts from recombinant study showed a profile of lineage marker that is identical to that of the de novo iBRAF* PCA tumors.
(1.37 MB TIF)

Figure S4 iBRAF* tumors regress after castration. A. Representative consecutive multi-slice MRI images (1.2 mm thickness) of pelvis of iBRAF* mouse #46 at baseline imaging (pre-castration) showing heterogeneous signal intensity characteristic of tumor (blue highlight). B. Changes in prostate tumor volumes over time after castration (n = 5) were calculated based on ROI on serial MRI images. For comparison, serial imaging of a WT mouse was shown. Asterisks indicate two post-castration tumors containing prostatic tumor cells on histological examination.
(1.37 MB TIF)

Figure S5 Activation of B-RAF pathway is not required for tumor progression and maintenance. A. Schematic representation of CI-1040 (a MEK inhibitor) treatment protocol using tissue recombination. Tissue recombinants were generated with iBRAF* prostate cancer cells and rat mesenchymal cells, implanted under the kidney capsule of nude mice, and grown for 2 months. The mice were orally treated with CI-1040 at 150 mg/kg body weight twice a day for two weeks. B. Gross morphology and graft weight after two-week treatment showed significantly increased graft size with CI-1040 treatment, compared to mock-treated control (p = 0.032). C. Histological analyses of grafts from mock-treated and CI-1040-treated mice confirmed prostate tumor development. Although decreased p-ERK staining with CI-1040-treated mice indicated the inhibition of B-RAF pathway, tumors were still proliferating, as manifested by strong positivity with Ki67 staining.
(1.37 MB TIF)

Figure S6 Strong activation of p-ERK and p-S6K was also observed in human prostate tumors harboring BRAFV600E mutation (total 8 samples; n = 4 with WT BRAF and n = 4 with BRAFV600E mutation). Importantly, two of the four human prostate tumors harboring BRAFV600E mutation showed no activation (BRAF V600 #S04-7014) or very weak activation of p-AKT (BRAF V600 #S04-7989).
(1.37 MB TIF)

## References

[1] Ellwood-Yen K, Graeber TG, Wongvipat J, Iruela-Arispe ML, Zhang J (2003). Myc-driven murine prostate cancer shares molecular features with human prostate tumors.. Cancer Cell.

[2] Hill R, Song Y, Cardiff RD, Van Dyke T (2005). Heterogeneous tumor evolution initiated by loss of pRb function in a preclinical prostate cancer model.. Cancer Res.

[3] Kim MJ, Bhatia-Gaur R, Banach-Petrosky WA, Desai N, Wang Y (2002). Nkx3.1 mutant mice recapitulate early stages of prostate carcinogenesis.. Cancer Res.

[4] Trotman LC, Niki M, Dotan ZA, Koutcher JA, Di Cristofano A (2003). Pten dose dictates cancer progression in the prostate.. PLoS Biol.

[5] Wang L, Cunningham JM, Winters JL, Guenther JC, French AJ (2003). BRAF mutations in colon cancer are not likely attributable to defective DNA mismatch repair.. Cancer Res.

[6] Mulholland DJ, Dedhar S, Wu H, Nelson CC (2006). PTEN and GSK3beta: key regulators of progression to androgen-independent prostate cancer.. Oncogene.

[7] Xin L, Teitell MA, Lawson DA, Kwon A, Mellinghoff IK (2006). Progression of prostate cancer by synergy of AKT with genotropic and nongenotropic actions of the androgen receptor.. Proc Natl Acad Sci U S A.

[8] Abate-Shen C, Shen MM (2000). Molecular genetics of prostate cancer.. Genes Dev.

[9] Bentel JM, Tilley WD (1996). Androgen receptors in prostate cancer.. J Endocrinol.

[10] Bookstein R, MacGrogan D, Hilsenbeck SG, Sharkey F, Allred DC (1993). p53 is mutated in a subset of advanced-stage prostate cancers.. Cancer Res.

[11] Brewster SF, Oxley JD, Trivella M, Abbott CD, Gillatt DA (1999). Preoperative p53, bcl-2, CD44 and E-cadherin immunohistochemistry as predictors of biochemical relapse after radical prostatectomy.. J Urol.

[12] Chen Z, Trotman LC, Shaffer D, Lin HK, Dotan ZA (2005). Crucial role of p53-dependent cellular senescence in suppression of Pten-deficient tumorigenesis.. Nature.

[13] Navone NM, Labate ME, Troncoso P, Pisters LL, Conti CJ (1999). p53 mutations in prostate cancer bone metastases suggest that selected p53 mutants in the primary site define foci with metastatic potential.. J Urol.

[14] Navone NM, Troncoso P, Pisters LL, Goodrow TL, Palmer JL (1993). p53 protein accumulation and gene mutation in the progression of human prostate carcinoma.. J Natl Cancer Inst.

[15] Stackhouse GB, Sesterhenn IA, Bauer JJ, Mostofi FK, Connelly RR (1999). p53 and bcl-2 immunohistochemistry in pretreatment prostate needle biopsies to predict recurrence of prostate cancer after radical prostatectomy.. J Urol.

[16] Theodorescu D, Broder SR, Boyd JC, Mills SE, Frierson HF (1997). p53, bcl-2 and retinoblastoma proteins as long-term prognostic markers in localized carcinoma of the prostate.. J Urol.

[17] Gioeli D, Mandell JW, Petroni GR, Frierson HF, Weber MJ (1999). Activation of mitogen-activated protein kinase associated with prostate cancer progression.. Cancer Res.

[18] Voeller HJ, Wilding G, Gelmann EP (1991). v-rasH expression confers hormone-independent in vitro growth to LNCaP prostate carcinoma cells.. Mol Endocrinol.

[19] Thomas RK, Baker AC, Debiasi RM, Winckler W, Laframboise T (2007). High-throughput oncogene mutation profiling in human cancer.. Nat Genet.

[20] Konishi N, Hiasa Y, Tsuzuki T, Tao M, Enomoto T (1997). Comparison of ras activation in prostate carcinoma in Japanese and American men.. Prostate.

[21] Lin S, Sahai A, Chugh SS, Pan X, Wallner EI (2002). High glucose stimulates synthesis of fibronectin via a novel protein kinase C, Rap1b, and B-Raf signaling pathway.. J Biol Chem.

[22] Sasaki Y, Niu C, Makino R, Kudo C, Sun C (2004). BRAF point mutations in primary melanoma show different prevalences by subtype.. J Invest Dermatol.

[23] Shiraishi T, Muneyuki T, Fukutome K, Ito H, Kotake T (1998). Mutations of ras genes are relatively frequent in Japanese prostate cancers: pointing to genetic differences between populations.. Anticancer Res.

[24] Suzuki H, Aida S, Akimoto S, Igarashi T, Yatani R (1994). State of adenomatous polyposis coli gene and ras oncogenes in Japanese prostate cancer.. Jpn J Cancer Res.

[25] Cho NY, Choi M, Kim BH, Cho YM, Moon KC (2006). BRAF and KRAS mutations in prostatic adenocarcinoma.. Int J Cancer.

[26] Gao H, Ouyang X, Banach-Petrosky WA, Gerald WL, Shen MM (2006). Combinatorial activities of Akt and B-Raf/Erk signaling in a mouse model of androgen-independent prostate cancer.. Proc Natl Acad Sci U S A.

[27] Tomlins SA, Rhodes DR, Perner S, Dhanasekaran SM, Mehra R (2005). Recurrent fusion of TMPRSS2 and ETS transcription factor genes in prostate cancer.. Science.

[28] Abate-Shen C, Shen MM (2002). Mouse models of prostate carcinogenesis.. Trends Genet.

[29] Kasper S (2005). Survey of genetically engineered mouse models for prostate cancer: analyzing the molecular basis of prostate cancer development, progression, and metastasis.. J Cell Biochem.

[30] Di Cristofano A, De Acetis M, Koff A, Cordon-Cardo C, Pandolfi PP (2001). Pten and p27KIP1 cooperate in prostate cancer tumor suppression in the mouse.. Nat Genet.

[31] Gao H, Ouyang X, Banach-Petrosky W, Borowsky AD, Lin Y (2004). A critical role for p27kip1 gene dosage in a mouse model of prostate carcinogenesis.. Proc Natl Acad Sci U S A.

[32] Majumder PK, Yeh JJ, George DJ, Febbo PG, Kum J (2003). Prostate intraepithelial neoplasia induced by prostate restricted Akt activation: the MPAKT model.. Proc Natl Acad Sci U S A.

[33] Kasper S, Tu W, Roberts RL, Shappell SB (2003). Transgenic mouse models for prostate cancer. Identification of an androgen-dependent promoter and creation and characterization of the long probasin promoter-Large T antigen (LPB-Tag) model.. Methods Mol Med.

[34] Zhang BH, Tang ED, Zhu T, Greenberg ME, Vojtek AB (2001). Serum- and glucocorticoid-inducible kinase SGK phosphorylates and negatively regulates B-Raf.. J Biol Chem.

[35] Chin L, Tam A, Pomerantz J, Wong M, Holash J (1999). Essential role for oncogenic Ras in tumour maintenance.. Nature.

[36] Tief K, Schmidt A, Beermann F (1997). Regulation of the tyrosinase promoter in transgenic mice: expression of a tyrosinase-lacZ fusion gene in embryonic and adult brain.. Pigment Cell Res.

[37] Regales L, Giraldo P, Garcia-Diaz A, Lavado A, Montoliu L (2003). Identification and functional validation of a 5′ upstream regulatory sequence in the human tyrosinase gene homologous to the locus control region of the mouse tyrosinase gene.. Pigment Cell Res.

[38] Hudson DL (2004). Epithelial stem cells in human prostate growth and disease.. Prostate Cancer Prostatic Dis.

[39] Verhagen AP, Ramaekers FC, Aalders TW, Schaafsma HE, Debruyne FM (1992). Colocalization of basal and luminal cell-type cytokeratins in human prostate cancer.. Cancer Res.

[40] Segawa N, Mori I, Utsunomiya H, Nakamura M, Nakamura Y (2001). Prognostic significance of neuroendocrine differentiation, proliferation activity and androgen receptor expression in prostate cancer.. Pathol Int.

[41] Cunha GR, Donjacour AA, Cooke PS, Mee S, Bigsby RM (1987). The endocrinology and developmental biology of the prostate.. Endocr Rev.

[42] Ma L, Teruya-Feldstein J, Bonner P, Bernardi R, Franz DN (2007). Identification of S664 TSC2 phosphorylation as a marker for extracellular signal-regulated kinase mediated mTOR activation in tuberous sclerosis and human cancer.. Cancer Res.

[43] Uzgare AR, Isaacs JT (2004). Enhanced redundancy in Akt and mitogen-activated protein kinase-induced survival of malignant versus normal prostate epithelial cells.. Cancer Res.

[44] Ji H, Wang Z, Perera SA, Li D, Liang MC (2007). Mutations in BRAF and KRAS converge on activation of the mitogen-activated protein kinase pathway in lung cancer mouse models.. Cancer Res.

[45] Aguirre AJ, Bardeesy N, Sinha M, Lopez L, Tuveson DA (2003). Activated Kras and Ink4a/Arf deficiency cooperate to produce metastatic pancreatic ductal adenocarcinoma.. Genes Dev.

[46] Byers RJ, Di Vizio D, O'Connell F, Tholouli E, Levenson RM (2007). Semiautomated multiplexed quantum dot-based in situ hybridization and spectral deconvolution.. J Mol Diagn.

[47] Tholouli E, Hoyland JA, Di Vizio D, O'Connell F, Macdermott SA (2006). Imaging of multiple mRNA targets using quantum dot based in situ hybridization and spectral deconvolution in clinical biopsies.. Biochem Biophys Res Commun.

