# Supplementary material for: BRAF Activation Initiates but Does Not Maintain Invasive Prostate Adenocarcinoma
Source: PLoS One. 2008 Dec 16;3(12):e3949. doi: 10.1371/journal.pone.0003949 (PMC2597248; doi:10.1371/journal.pone.0003949)
Supplement: Table S1 — (0.03 MB RTF) [file pone.0003949.s001.rtf]

Supplemental Table 1. The summary of phenotypes of iBRAF* transgenic male mice 	
A. Line 29	
Number	Mouse I. D.	Sex	Genotype	Age of deathc (weeks)	Doxy	Pheotypesd	
1	233	M	iBRAF* a	25	off	no obvious tumors	
2	238	M	iBRAF*	34	off	lymphoma	
3	239	M	iBRAF*	10	off	no obvious tumors	
4	240	M	iBRAF*	29	off	no obvious tumors	
5	241	M	iBRAF*	34	off	lymphoma	
6	423	M	iBRAF*	33	off	sarcoma	
7	531	M	iBRAF*	37	off	no obvious tumors	
8	547	M	iBRAF*	24	off	lymphoma	
9	762	M	iBRAF*	39	off	no obvious tumors	
10	662	M	Tet-BRAF* b	35	on	lymphoma	
11	731	M	Tet-BRAF*	43	on	sarcoma	
12	747	M	Tet-BRAF*	30	on	no obvious tumors	
13	700	M	Tet-BRAF*	47	on	lymphoma	
14	668	M	Tet-BRAF*	50	on	sarcoma	
15	761	M	Tet-BRAF*	14	on	no obvious tumors	
16	794	M	Tet-BRAF*	32	on	no obvious tumors	
17	862	M	Tet-BRAF*	36	on	sarcoma	
18	863	M	Tet-BRAF*	33	on	sarcoma	
19	919	M	Tet-BRAF*	16	on	no obvious tumors	
20	61	M	iBRAF*	37	on	prostate cancer	
21	65	M	iBRAF*	32	on	lymphoma	
22	76	M	iBRAF*	25	on	prostate cancer	
23	86	M	iBRAF*	23	on	prostate cancer	
24	102	M	iBRAF*	32	on	prostate cancer	
25	111	M	iBRAF*	18	on	lymphoma and sarcoma	
26	113	M	iBRAF*	34	on	prostate cancer	
27	137	M	iBRAF*	26	on	prostate cancer	
28	139	M	iBRAF*	29	on	prostate cancer	
29	260	M	iBRAF*	32	on	prostate cancer	
30	516	M	iBRAF*	14	on	prostate cancer 	
31	558	M	iBRAF*	22	on	melanoma	
32	565	M	iBRAF*	22	on	lymphoma	
33	584	M	iBRAF*	22	on	prostate cancer	
34	600	M	iBRAF*	16	on	no obvious tumors	
35	620	M	iBRAF*	21	on	lymphoma	
36	623	M	iBRAF*	31	on	melanoma	
37	654	M	iBRAF*	19	on	prostate cancer 	
38	831	M	iBRAF*	16	on	prostate cancer	
39	832	M	iBRAF*	16	on	prostate cancer	
40	834	M	iBRAF*	13	on	no obvious tumors	
41	835	M	iBRAF*	20	on	prostate cancer	
42	839	M	iBRAF*	25	on	prostate cancer	
43	749	M	iBRAF*	16	on	prostate cancer	
44	898	M	iBRAF*	24	on	prostate cancer	
45	899	M	iBRAF*	27	on	no obvious tumors	
46	900	M	iBRAF*	23	on	no obvious tumors	
47	913	M	iBRAF*	20	on	prostate cancer	
48	905	M	iBRAF*	32	on	prostate cancer	
49	908	M	iBRAF*	32	on	prostate cancer	
50	920	M	iBRAF*	25	on	no obvious tumors	
51	914	M	iBRAF*	29	on	prostate cancer	
52	910	M	iBRAF*	30	on	lymphoma	
B. Line 13	
Number	 	Sex	Genotype	Age of death(weeks)	Doxy	Pheotypes 	
1	226	M	iBRAF*	13	on	no obvious tumors	
2	248	M	iBRAF*	11	on	no obvious tumors	
3	294	M	iBRAF*	5	on	no obvious tumors	
4	346	M	iBRAF*	17	on	prostate cancer	
5	347	M	iBRAF*	38	on	melanoma	
6	350	M	iBRAF*	22	on	prostate cancer	
7	419	M	iBRAF*	28	on	melanoma	
8	423	M	iBRAF*	4	on	no obvious tumors	
9	450	M	iBRAF*	30	on	prostate cancer	
10	539	M	iBRAF*	36	on	sarcoma	
a  Tet-BrafE600+, Tyr-rtTA+, Ink4a/Arf -/-	
b  Tet-BrafE600+, Tyr-rtTA-, Ink4a/Arf -/-	
c  Age of the mouse that had been sacrificed for the detailed histological examinations due to the visible tumor   formation or sickness.   	
	
d   Each tumor type was determined by histological examination.	
