# Supplementary figures and images for: BRAF Activation Initiates but Does Not Maintain Invasive Prostate Adenocarcinoma
Source: PLoS One. 2008 Dec 16;3(12):e3949. doi: 10.1371/journal.pone.0003949 (PMC2597248; doi:10.1371/journal.pone.0003949)

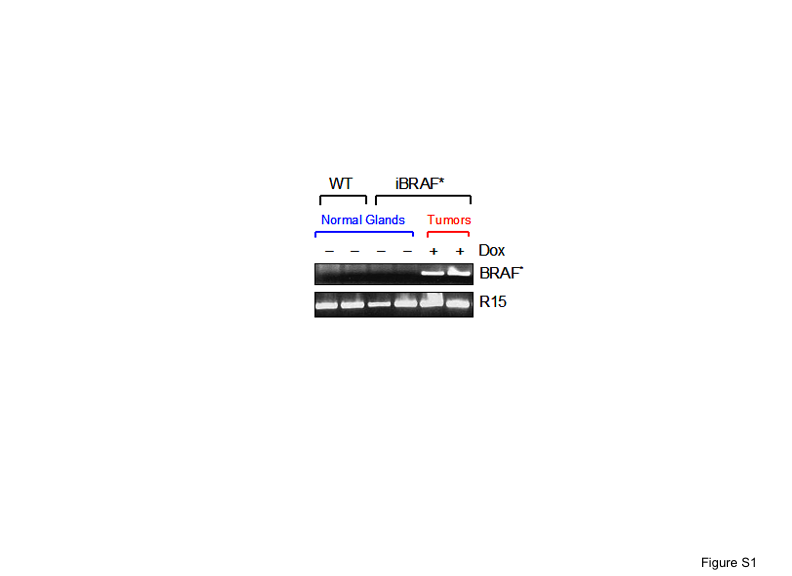

Supplement: Figure S1 — BRAF* transgene expression is documented by transgene-specific RT-PCR with two independent prostate tumors of bi-transgenic iBRAF* mice on doxycycline. (1.37 MB TIF) [file pone.0003949.s002.tif]

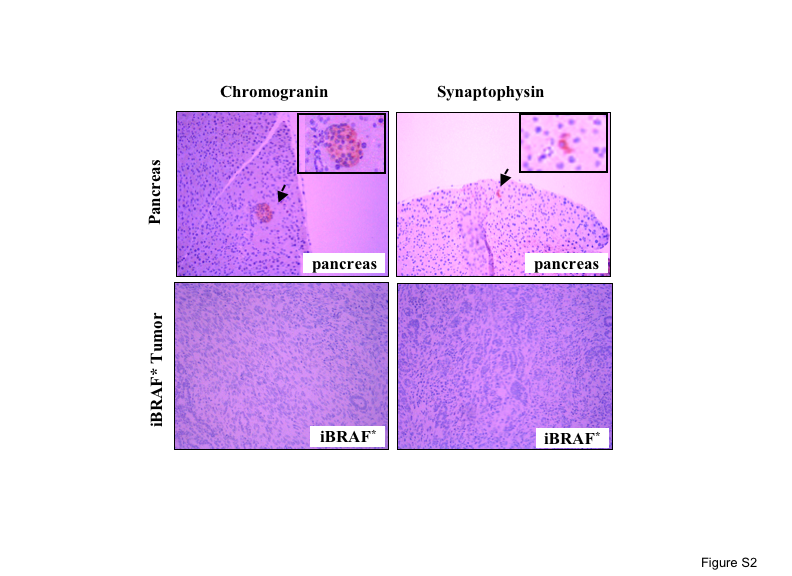

Supplement: Figure S2 — Both ductal and spindled components of iBRAF* prostate tumors were negative for neuroendocrine markers (chromogranin and synaptophysin) by IHC. For controls, pancreatic tissues were used. (1.37 MB TIF) [file pone.0003949.s003.tif]

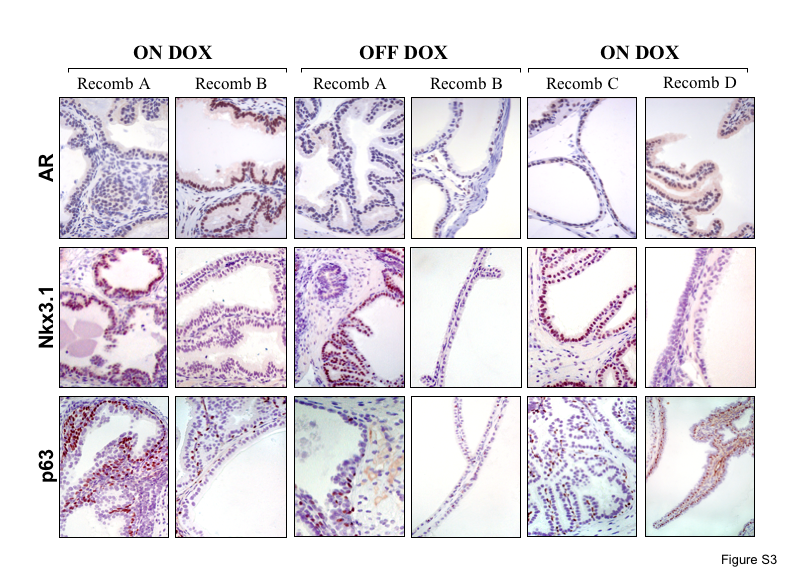

Supplement: Figure S3 — Grafts from recombinant study showed a profile of lineage marker that is identical to that of the de novo iBRAF* PCA tumors. (1.37 MB TIF) [file pone.0003949.s004.tif]

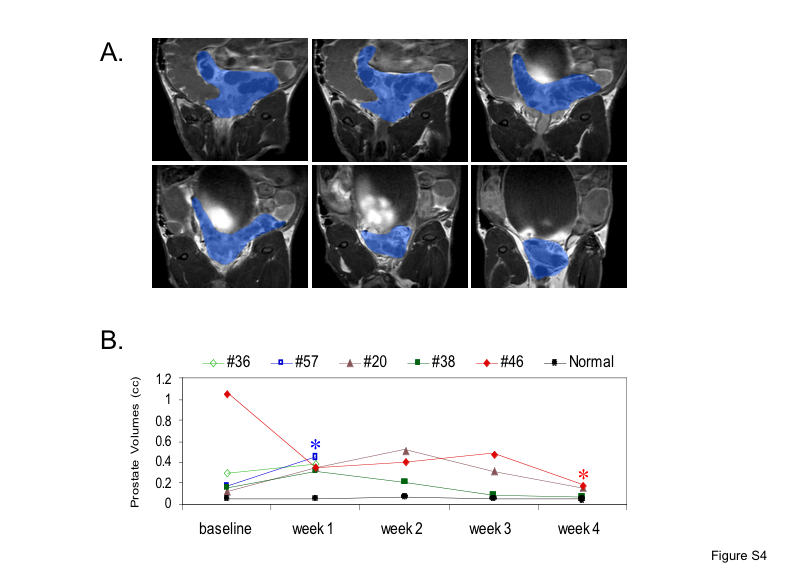

Supplement: Figure S4 — iBRAF* tumors regress after castration. A. Representative consecutive multi-slice MRI images (1.2 mm thickness) of pelvis of iBRAF* mouse #46 at baseline imaging (pre-castration) showing heterogeneous signal intensity characteristic of tumor (blue highlight). B. Changes in prostate tumor volumes over time after castration (n = 5) were calculated based on ROI on serial MRI images. For comparison, serial imaging of a WT mouse was shown. Asterisks indicate two post-castration tumors containing prostatic tumor cells on histological examination. (1.37 MB TIF) [file pone.0003949.s005.tif]

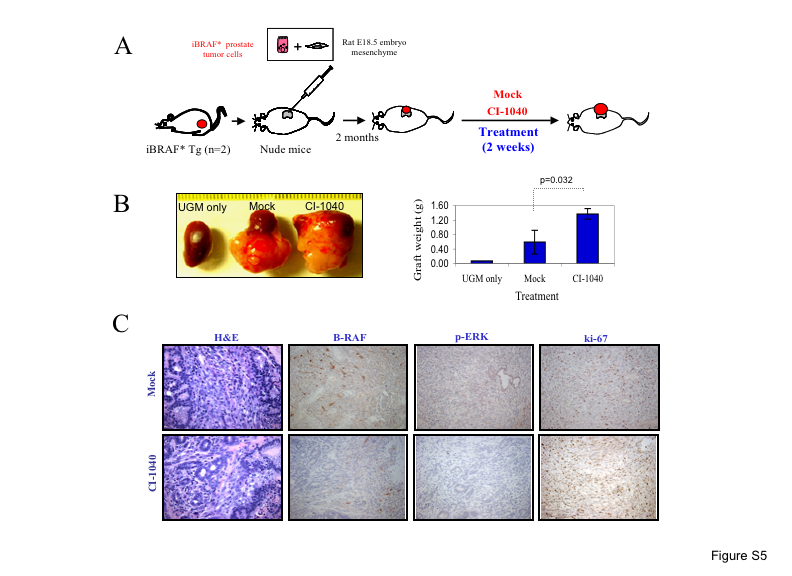

Supplement: Figure S5 — Activation of B-RAF pathway is not required for tumor progression and maintenance. A. Schematic representation of CI-1040 (a MEK inhibitor) treatment protocol using tissue recombination. Tissue recombinants were generated with iBRAF* prostate cancer cells and rat mesenchymal cells, implanted under the kidney capsule of nude mice, and grown for 2 months. The mice were orally treated with CI-1040 at 150 mg/kg body weight twice a day for two weeks. B. Gross morphology and graft weight after two-week treatment showed significantly increased graft size with CI-1040 treatment, compared to mock-treated control (p = 0.032). C. Histological analyses of grafts from mock-treated and CI-1040-treated mice confirmed prostate tumor development. Although decreased p-ERK staining with CI-1040-treated mice indicated the inhibition of B-RAF pathway, tumors were still proliferating, as manifested by strong positivity with Ki67 staining. (1.37 MB TIF) [file pone.0003949.s006.tif]

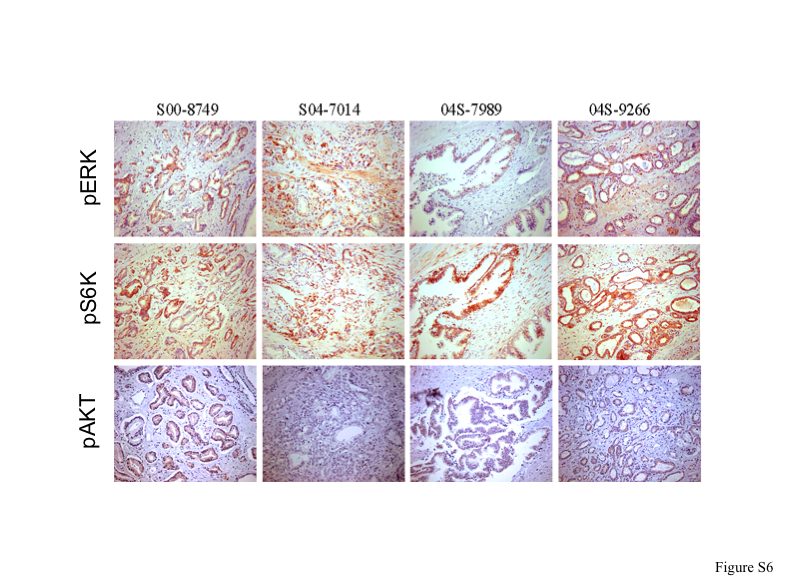

Supplement: Figure S6 — Strong activation of p-ERK and p-S6K was also observed in human prostate tumors harboring BRAFV600E mutation (total 8 samples; n = 4 with WT BRAF and n = 4 with BRAFV600E mutation). Importantly, two of the four human prostate tumors harboring BRAFV600E mutation showed no activation (BRAF V600 #S04-7014) or very weak activation of p-AKT (BRAF V600 #S04-7989). (1.37 MB TIF) [file pone.0003949.s007.tif]
